# Basis for a Qualitative Research Project: The Research Context

**DOI:** 10.17533/udea.iee.v44n1e15

**Published:** 2026-03-31

**Authors:** Carmen de la Cuesta Benjumea

**Affiliations:** 1 Nurse, Ph.D. Honorary Collaborating Professor, Department of Health Psychology. University of Alicante, San Vicente del Raspeig, Spain. Alicante Institute for Health and Biomedical Research (ISABIAL), Alicante, Spain. Email: ccuesta@ua.es https://orcid.org/0000-0003-2160-392X Universidad de Alicante Department of Health Psychology University of Alicante Alicante Spain ccuesta@ua.es

## Presentation

This issue of our journal, *Investigación y Educación en Enfermería,* marks the beginning of a series of articles from a comprehensive work on the bases for developing and executing a qualitative research project in health. The topics covered are the research context, an introduction to qualitative research, qualitative approaches, qualitative design, qualitative data generation, qualitative data analysis, quality and ethics in qualitative research, and finally, writing the qualitative report. Accompanying the theoretical content, each article presents examples of studies already carried out that illustrate the practice of what is being discussed. At the end, exercises are proposed to consolidate what has been learned. This promotes a reflective and active reading of the work.

The series is aimed primarily at young researchers. However, lecturers of qualitative research will find numerous examples in the texts that illustrate theoretical aspects and the practice of qualitative research in the field of health. This work will also be of interest to educators who want to familiarise themselves with qualitative research methodology. With this publication commitment, the Editorial Committee of the Journal of Nursing Research and Education is committed to quality research that is relevant to the field of health and care. This issue includes the first two articles of the work, entitled: The Context of Research and Introduction to Qualitative Research. 

### Basis for a qualitative research project: the research context

This first article begins a series that makes up the manuscript titled “Bases for developing and implementing qualitative research in health.” Reading them will allow you to start your study on the right foot. Once you have started, with experience and reflexivity, you will build a researcher’s self. The manuscript draws extensively on classic bibliographic sources, a heritage that we must preserve and pass on. I hope you will enjoy the reading and that it will inspire you. 

1. Ways of producing knowledge

Perhaps one of the most novel issues for those approaching qualitative research for the first time is the fact that there are different ways of inquiring about the world around us. All of them are legitimate and provide useful knowledge for acting in this world. Research is about knowledge construction, which serves two purposes: one is to predict the occurrence of events, and the other is to explain or make them clear. These distinctions should not be taken too rigidly, although the meaning of explanation should be examined. In his essay entitled "Explanation and Understanding," von Wright distinguishes between two traditions of thought that disagree on the conditions that must be met for an explanation to be scientifically respectable.^1^ One has been described as Aristotelian and supports the teleological or conceptual view of explanation, while the other is Galilean, where explanation is causal, formulated in terms of laws that link phenomena. These two traditions represent fundamentally opposing philosophies of the scientific method: positivism, which rose to prominence in the mid-19th century, and anti-positivism, which emerged in the late 19th and early 20th centuries. Over the last few centuries, both have alternated their influence on the development of science ^1^ Three principles stand out in positivist philosophy. The first is the principle of methodological monism which considers that there is only one scientific method. The second establishes that the canon or methodological ideal is represented by the natural sciences, where objectivity is understood as neutrality, research is value-free, and data is collected by a researcher who is external or separate from what they are investigating.^2^ The third principle conceives of causal explanation in a broad sense and relies on general laws such as subsumption to achieve its goal of generalising reproducible and predictable phenomena. These principles link positivist philosophy to the Galilean position. ^1^

**Figure 1:**
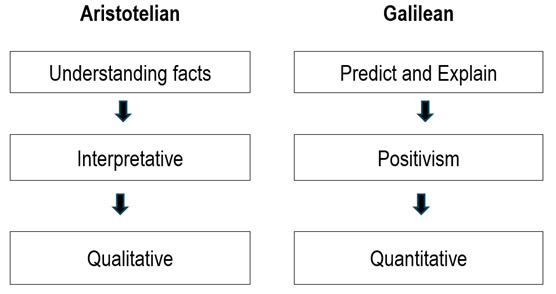
Traditions in knowledge production

On the contrary, the anti-positivist philosophy of science, known as hermeneutics, rejects methodological monism, making it more diverse and heterogeneous regarding method, as we shall see in the third article of the series. Furthermore, it rejects the methodological canon of the natural and exact sciences and considers that neutrality cannot be achieved, since the observation of something depends on the theory from which it is observed. On the other hand, it argues that the neutrality of the researcher is not relevant in the study of human beings, since what matters is to capture subjectivity.^2^ Finally, in contrast to positivism's generalisation of reproducible and predictable phenomena, this hermeneutic philosophy seeks to understand their particularities and therefore challenges causal explanation.^1^ Thus, it is related to the Aristotelian position, which uses qualitative methods to achieve its aim of understanding the particularities of a phenomenon. Figure 1 summarises these two traditions.

There is an example in van Maanen's text ^3^ that illustrates very well the differences between these ways of seeing the world. In a seminar, one researcher begins by saying that many people are currently bored at work. Another researcher interrupts him to ask how many people are bored, why they are bored, how long they are bored, where they work, and where they come from. The qualitative researcher responds, ‘It's not important.’ While the first researcher is interested in the experience of boredom, the second is interested in knowing details about the people who are bored. The relevance of what one wants to know depends on the paradigmatic position of the person asking the question.

The fact that there are two ways of generating knowledge, one narrative and the other numerical, does not mean that one cancels out the other or that one is superior to the other. Rather, both contribute knowledge about the world, and which one is used will depend on what one wants to know. However, the maturity of science has been determined by quantification. ^(4^ Thus, those sciences that have paid attention to quantification have been called *strong* sciences, and those that are not or are less quantifiable have been called *soft* sciences. Although this terminology is now considered outdated, it is true that there are still remnants of discrimination regarding the legitimate way of producing scientific knowledge. For instance, science is usually defined as that produced by quantitative methods, and knowledge produced by qualitative methods is excluded from what is considered scientific. The cardinal issue, as von Wright explains, is not the method but the choice of method we make to produce science.^1^ The method, in turn, is intertwined with and emerges from disciplines and perspectives.^4^ Thus, in the social sciences, counter currents emerged opposing quantification for being irrelevant, and alternative paradigms for producing knowledge were proposed. Let us see what these are.

1.1. Four paradigms

**Figure 2:**
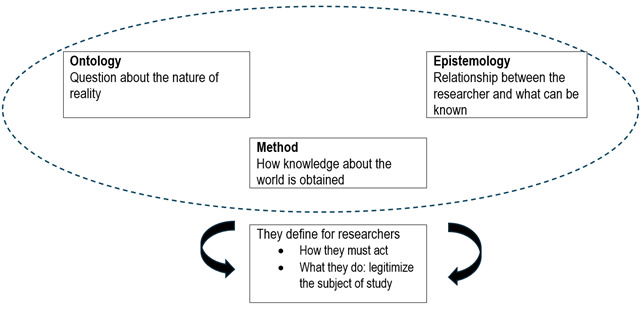
Interconnected elements of paradigms in social sciences

The social sciences and humanities emerged under the tension of positivist and anti-positivist trends in the last century. ^1^ Within them, different groups of beliefs known as paradigms can be distinguished, which determine what is researched and how it is done; paradigms explain and rationalise the differences between quantitative and qualitative methods. These paradigms are not open to verification in the classical sense, as they deal with beliefs about what reality is (ontology), what the relationship between the investigator and what is to be known should be (epistemology), and what the strategy for knowing should be (method).^4^ These issues are not independent, but are interconnected, as shown in Figure 2.

According to social sciences, four paradigms can be distinguished:^5^ 1) the positivist paradigm, which presents a traditional perspective that has dominated the physical and social sciences; 2) the post-positivist paradigm, which is a response, albeit limited, to the criticisms levelled at positivism, especially in the interwar period;^1^ 3) the critical paradigm, which brings together a series of alternative paradigms such as feminism, neo-Marxism and postmodernism, that have in common the view that research is governed by values; and 4) the constructivist paradigm, which basically understands that reality is not given but constructed. 

The paradigms are still in the process of formation; they are neither definitive nor compartmentalised as they influence each other. An example of this is that years after authors describing them, they added another one called participatory, which is made up of post-positivist and critical theory orientations.^4^ They also included a fourth value: axiological, which, referring to the researcher, answers questions of how to be a moral person in the world, so that each epistemology carries with it an ethical and moral stance on the world and on oneself. Therefore, this value places ethics within and not outside paradigms. Regardless of future developments and nuances, understanding the four original paradigms serves as an introduction to the diversity of research methods and as an antidote to the stance of a single scientific method. As an introduction, let us look at the main differences according to the three questions that shape them: the ontological, the epistemological, and the methodological.^5^ In doing so, I hope to trigger your curiosity and motivate you to study them in depth, because, as these authors assert, no researcher can afford to be oblivious to basic ontological, epistemological, and methodological assumptions. I will begin by presenting the recent constructivist and critical paradigms that inspired qualitative methods to contrast them with the other two.

*Ontology.* Ontology refers to what we believe reality is. The major difference between the constructivist paradigm (Also referred to as naturalistic, interpretative, phenomenological, or “other”) and the other three paradigms is that it understands reality as being constructed through people's actions and that its nature depends on the point of view taken. For example, a patient's experience at a gynaecological appointment will be different from that of the doctor treating her. This paradigm is based on what is called a relativistic ontology: reality is plural, there are multiple realities. Goffman reflects this very well when, in his study on the situation of the mentally ill patient, he states that in order to describe the patient's situation accurately, it is essential to present it from a partial perspective, since: ‘almost all professional work on the mentally ill has been written from the point of view of the psychiatrist who, speaking in social terms, is situated, from my perspective, on the opposite side’.^6^ It is interesting to note that Goffman, as a researcher, does not place himself outside, claiming a third point of view, but rather adopts that of the patient, which, as you will see below, addresses the paradigm epistemology. Thus, the cardinal issue in qualitative research will be to reveal a point of view and understand that it is created based on a concrete experience such as that of being mentally ill. Qualitative data will therefore be subjective and based on first-hand experiences.

The critical paradigm also considers that reality is constructed, but not through action, but through social values, power structures, and conflicts that crystallise over time. This paradigm represented a break with what had been understood about reality up to that point, introducing the idea that reality was historically formed, in such a way that it seemed inappropriately natural or real. This ontology is called historical realism. A very clear example of this is to consider health inequalities as products of unequal power relations and not of biology. Here, reality is not believed to be multiple as in the previous paradigm. 

The positivist and post-positivist paradigms also understand that reality is one, but unlike the critical paradigm, they see it as something that is given without human intervention. It is an objective reality that can be known through instruments. Post-positivism was a reaction to the positivist ideas of the 19th century and by considering that reality can only be known in an imperfect and probabilistic way, gave way from what was called the naive realism of positivism, to critical realism.

***Epistemology*.** Epistemology refers to the relationship that should exist between what is known and what is yet to be known. In the constructivist paradigm, knowledge is created between the researcher and the study participants, so there is no conventional separation between ontology and epistemology. Here, to generate knowledge, the researcher interacts directly with the study subjects. Epistemology is subjectivist because it is interested in knowing people's points of view, and it is transactional or exchange-based because it understands that the subjects of the study have knowledge that is of interest to the researcher. The knowledge produced in the study is created in the sense that it is generated between the researcher and the study participants. On the other hand, in critical theory, epistemology is like constructivism, although it emphasises that knowledge will be mediated by the researcher's values.

In positivism, epistemology is objectivist and dualistic, placing the researcher and the subjects on different levels, with a rigid and hierarchical division in which the researcher is a privileged expert. It assumes that the researcher and the subject of study are independent and that the researcher can study the subject without influencing it or contaminating it. In this paradigm, the knowledge produced is real in the sense that it is objective, can be apprehended by means of valid and reliable instruments, and is reproducible in other studies, i.e., following the same procedures will yield the same results. Post-positivism, on the other hand, abandons positivist dualism because it recognises the impossibility of a complete separation between the researcher and the subject of research, but maintains objectivism, albeit modified, emphasising the need for experts to act as guardians of objectivity in the research process and for the knowledge produced in the study to fit within pre-existing knowledge. Thus, in these studies, it will be relevant to confirm what has been found in previous studies. As in positivism, this knowledge is reproducible, although it recognises that it is only probably true, in addition, it is bound to being proven false. These views are a consequence of the ontological position of critical realism.

*Method.* The method deals with the procedures for acquiring knowledge. In the constructivist and critical paradigm, the method is dialectical. However, while constructivism uses hermeneutic techniques that are contrasted in a dialectical exchange with the subjects of the study, critical theory engages in a reasoned, principle-based dialogue that transforms the knowledge and consciousness of the subjects participating in the study. Qualitative methods ascribe to these paradigms. In the positivist paradigm, the method is experimental and being manipulated. Here, efforts are focused on verifying a hypothesis or proposition through mathematical formulas. The method basically consists of conducting an empirical test to verify a hypothesis. During this test, conditions are manipulated to prevent them from influencing the results. In post-positivism, the method remains experimental, but the manipulation of conditions is modified. Thus, it emphasises the plurality of criticism, that is, using multiple sources and mechanisms to prove the falsity of a hypothesis rather than to verify it. Since it seeks to rectify the problems of positivism, it may include qualitative techniques. Obviously, the mere presence of qualitative techniques does not mean that the study is qualitative; for this, its ontological and epistemological bases must be present. Table 1 presents a summary of the paradigmatic characteristics just discussed in the same order in which they have been presented here. The aim is to enable you to differentiate briefly between the different positions for generating knowledge, bearing in mind that paradigms are not sealed compartments, as shown by the dotted lines in the image.

**Table 1:** Scientific paradigms in social sciences

Element	Constructivism	Critical	Post-positivism	Positivism
Ontology	Realities are constructed through action and in a situated manner.	The reality shaped by social values that has become established over time.	A single "real" reality that can only be known imperfectly and probabilistically.	A single "real" reality that can be known by means of instruments.
Epistemology	Relationship between the researcher and the subjects of the study. Subjectivist. Findings are constructed during the research process.	Relationship between the researcher and the subjects of the study. Subjectivist. Findings are mediated by values.	Separation between subject and object. Modified objectivist. The results are probably true. .	Separation between subject and object of study. Objectivist. The results are repeatable and true.
Method	Interpretative and dialectical.	In dialogue between equals and dialectical.	Experimental-Manipulation modification-Testing the falsity of the hypothesis. May include qualitative techniques.	Experimental-Manipulación. Verificación de hipótesis. Métodos cuantitativos.

Fuente: Adapted from Guba y Lincoln 1994.

The importance of recognising these paradigmatic positions lies in the fact that they determine the validity of a study and legitimise the research question, as indicated at the bottom of the figure 2. Thus, when faced with the question of whether something can be researched, whether it is relevant, and how it should be done, the answer is: ‘it depends’ on what is believed about the nature of reality, of knowledge, and the appropriate way to access it. How valid a qualitative study may be, it will be tied to the paradigm of the researcher and, therefore, the legitimacy of its results. ^7^ However, a paradigm is not a position that we adopt according to the demands of each situation, as if it were a jacket to were. A paradigm is not chosen; rather, we acquire it and express it in the approaches we take to what we study and how we will carry it out. As researchers, we are constantly learning and refining our skills through practice. Our inclination towards how we see the world and question it, is developed through contact with mentors, reading and experience. Likewise, as von Wright points out, it also implies that we make existential decisions that cannot be justified a posteriori^1^.

1.2 Interpretive frameworks

**Figure 3:**
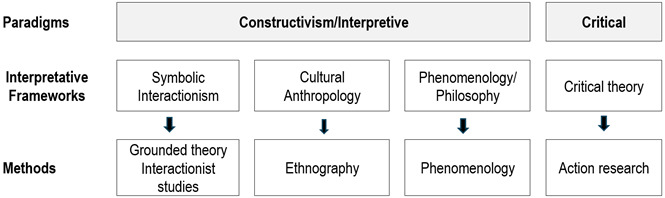
Paradigm, interpretative framework, method

In addition to paradigms, there are schools of thought that serve as a lens for approaching the study, interpreting the data, and developing the discussion of the findings. ^8^ They can also inform the choice of research method. Together with the paradigm, shape the qualitative gaze. Below, I present four schools of thought or interpretative frameworks that have given rise to four qualitative methods with wide-ranging implications in the field of health. These are: symbolic interactionism, cultural anthropology, phenomenology, and critical theory. First, I outline their most relevant characteristics and then indicate the qualitative research method they have given rise to, followed by examples of studies undertaken. These are high-quality studies and therefore a valuable resource for your learning. To help you understand, Figure 3 presents the relationship between paradigm, interpretative framework and method.

### Symbolic interactionism

Symbolic interactionism is a sociological movement that originated in the Chicago school in the 1960s. The roots of interactionism lie in pragmatism and the criticism of psychological behaviourism articulated in the 1920s and 1930s. ^9^ Pragmatists believe that reality does not exist outside the world but is created when people act in and towards it, and this action is the basis for understanding their behaviour. For pragmatists, reality is indeterminate and fluid, and therefore open to multiple interpretations. What is real are the consequences, as Thomas' theorem points out ^9^: If people define situations as real, they are real in their consequences. Critics of psychological behaviourism at the time argued that human behaviour cannot be explained solely by its visible aspects, but that there is a hidden thought process involving symbols and meanings. ^9^ Interactionist theory, therefore, focuses on the mental capacities of the individual, who is seen as an actor in the social world. 

Herbert Blumer articulates the interactionist perspective in his 1969 book. Here he expounds on the importance of the meaning that people attribute to their social interactions and how this shapes their behaviour. For Blumer, meaning is not derived from mental processes but from the process of interaction with other people^9^. Interactionism considers that people are involved in a continuous process of problem solving and are shaping their environment. To do this, they act by interpreting their environment and the stimuli they receive from it. From this perspective, the stimuli that people receive are not commands to act, but opportunities to do so. Whether they act or not depends on how they judge or understand their situation, ^10^ that is: the meaning it has for them.

The premises that best summarise Blumer's interactionism are:^10^ 1) Human beings act on things based on the meaning they have for them; 2) The meaning of these things is derived or emerges from social interaction; and 3) These meanings are used and transformed through an interpretive process as people encounter situations. Thus, interactionism assumes that behaviour should be understood as a process in which people shape and control their behaviour by considering the expectations of others with whom they interact. Therefore, symbolic interactionists are interested in the interpretive process that people engage in during their daily lives and in clarifying their behaviour as result of this interpretative process. ^11^ Here, language, composed of symbols whose meaning should not be taken for granted, is the object of research. Words such as medicine, medical error, and healthcare are worthy of study because their meaning varies depending on the context in which they are used. For interactionists, the observation of interactions, conversations, and documents lay out, are worthy of research. For example, Millman observed the medical profession for two years in different settings such as emergency rooms and operating theatres, mortality committees, and staff meetings. Based on this observation, she described a variety of ways in which doctors define, perceive, and respond to medical errors. She found that to justify errors, beliefs such as ‘every case is unique’ and ‘medicine is an art, not a science’, were used. ^12^

Symbolic interactionism contributed to the development of the grounded theory method and is a good interpretative framework for studies that seek to understand human behaviour in a non-deterministic way and how it unfolds over time. In the area of health, in addition to Millman's study, Howard Becker and colleagues'^13^ study on the socialisation of medical students, stands out in which the experiences and interactions of students with their teachers in class and in clinical settings are made visible and analysed. Also noteworthy is David Sudnow's leading work on the social organisation of death in hospitals. In this study, he considers that categories such as patient, dying and death are constituted by the practices of the staff. In his words: ‘death and dying constitute, from this [interactionist] perspective, the series of practices performed by the team [hospital staff] when they use these terms in the course of their daily work in the hospital wards’^14^ In short, interactionism has served as a guide for formulating the research question, developing the study design, and directing data generation in various qualitative studies^15^

### Cultural anthropology

Culture is the focus of anthropology and can be defined as a group's way of life. One of the significant characteristics of culture is its transmission through social rather than biological mechanisms, so that culture is a form of social inheritance. ^16^ People in a culture share values and ideas that they learn from other members of the group. Culture is an organised system composed of three interrelated subsystems: technological, sociological and ideological. ^16^ The technological subsystem consists of instruments and the techniques used to operate them. These include, among other things, production tools, means of subsistence, and shelter materials. The sociological subsystem, for its part, consists of interpersonal relationships that are expressed in individual and collective behaviour. These include, for example, kinship, economic, political, occupational, and professional networks. Finally, the ideological sphere is composed of ideas, beliefs and knowledge that are expressed in spoken or symbolic language and are formed, among other things, by mythologies and theologies, legends, science and popular knowledge.

The ethnographic method is used to study ways of life or cultures. Its conceptual basis and how it is carried out reflect the characteristics of cultural anthropology. However, it is difficult to separate the ethnographic method from its interpretative framework, since culture as such ‘does not exist until someone acting in the role of ethnographer places it there’.^17^ In other words, culture is attributed by the ethnographer. I believe it is necessary to clarify that culture is not limited to a physical space shared by a group, but also encompasses those people who, although physically separated, share an experience. Because of this, they share values and ways of life, or experiences such as being the mother of a child with cancer or suffering from a chronic illness such as multiple chemical sensitivity (MCS). Many qualitative studies have used cultural anthropology as an interpretative framework. It is worth mentioning the work on reproductive and child health, ageing and mental health, among others, collected in the text Anthropology and Nursing. ^18^ and Lipson's significant work on people with MCS. This study shows that, as there is no cure, people with MCS manage their symptoms through prevention and avoidance of chemicals, detoxification and emotional self-care. ^19^

### Phenomenology

Phenomenology derives from early 20th-century German philosophy, which was interested in understanding how people become aware of their experiences. Today, phenomenology is a variety of interrelated philosophies, notably Husserl's eidetic or descriptive philosophy and Heidegger's hermeneutic philosophy. ^20^ Husserl's eidetic philosophy is epistemological, as it deals with the nature and foundations of knowledge. Its purpose is to make experience evident through direct perception or clear intuition, using descriptive language. Heidegger's hermeneutic philosophy, on the other hand, is ontological, as it deals with the way of being in the world. He understands that truth is found in the interpreted world and thus his goal is to discover the meaning that is not immediately apparent to our intuition. Hermeneutic philosophers seek to understand and therefore must go beyond what is directly perceived.^20^ In hermeneutics, there are four key existential themes: 1) Corporeality, which refers to the lived body, which may be normal or distorted, for example, due to illness; 2) Relatedness, which refers to the lived other, that is, relationships with others and with the world in general; 3) Spatiality, which refers to the space in which we live; and 4) Temporality, which deals with lived time, time as experienced.

While for Husserl phenomenology is everything: a philosophy, an approach and a method of inquiry, for Heidegger it is not, and he develops phenomenology into interpretative or hermeneutic methods. Thus, what we refer to here as phenomenology refers to a philosophy that serves as an interpretative framework in research and to a qualitative method whose purpose is to capture the lived experience which should not be confused with philosophy or a variety of philosophies. This method of inquiry has in turn been developed in different ways by different schools, such as that of Utrecht. ^20^ In the field of health, studies using hermeneutic phenomenology as an interpretative framework and method are common. Noteworthy examples include Madjar's work on clinically inflicted pain, which describes the lived experience of pain caused in the context of medical treatment,^21^ and Castillo and Chesla's study on the feelings, expectations and everyday concerns of parents of children with leukaemia.^22^ In this study, they found that at first, parents experienced their children's cancer as a catastrophe that put an end to their world, but gradually they learned to live with a disease familiar to them, albeit treacherous.

### Socio-critical theory

This interpretative framework emerges from the work of early 20th-century German philosophers and economists who investigated power relations and sought social transformation. Socio-critical theory reveals the political and social agendas that shape, limit, and influence people's lives.^23^ Marx's work paved the way for the development of critical theory, and he is considered the first to use the critical method in the social sciences by focusing his work on the notion of critique.^24^ In his writings, Marx suggested that both social scientists and vulnerable people should fight for social change. Consequently, this interpretative framework uses research to interpret or promote social action, to empower people so that they can overcome barriers of class, race and gender.^7^ Among the beliefs of socio-critical theory, it is emphasised that ideology guides and shapes research, that researchers must question and examine power structures in their studies, and that people must participate in a dialogue of equals in the search for truth.^24^

Socio-critical theory shapes the participatory action research method and conditions the research topics that critical researchers choose, including those related to historical problems of domination, social struggles, and criticism of society^7^. However, it should be the people affected by marginalisation and oppression who ultimately define the topics to be researched, not external experts. ^23^ In the field of health, issues such as inequalities, power structures within the system, professional training, and community participation have been addressed. For example, one study used this theory as a lens to reveal issues of marginalisation, oppression, racism, gender, and discrimination in a clinic for the treatment of heroin addiction, ^25^ This work provided a better understanding of the impact of stigma on clinic users and the power exercised over them. Of note is the outstanding study on environmental conflict in the Huasco Valley in Chile, in which the researcher took a critical look at the social movement against the adverse effects of industry on the health of the Valley's population. ^26^

2. Concluding remarks

In this article, I have argued that paradigms are not perspectives that are adopted on an ad hoc basis for the development of a study, but rather they are the ways in which researchers view the world they are inquiring and act accordingly. Given that as researchers we are not separate from the research process itself, but rather we are part of it, recognising one's own perspective is fundamental in qualitative research. In the field of health, the positivist mentality dominates, that is, the objective way of seeing things, in which the important issue is to measure the phenomenon without questioning its nature. Health professionals have been socialised in this perspective, ^27^ and as Lincoln ^28^ shows, it is not sufficient, nor sometimes adequate, to deal with health issues, as these are tied to people's subjectivity. Qualitative research provides a unique insight into the emotional, social, cultural and contextual aspects of health and illness. Fortunately, there are increasingly greater and better approaches between methods and researchers. The so-called wars of paradigms are being left behind, and the colonisation of one method over another is fruitless. In the article that follows, I focus on the characteristics of the qualitative method, which are determined by the paradigm on which it is based and which serve as the method's hallmarks. Likewise, to provide a context for learning free of possible misunderstandings, I explore the meanings of terms and procedures that are central to qualitative studies.

3. Exercise

The aim of this exercise is for you to identify your ideas or preconceptions about research. Being aware of them is the first step in your learning about qualitative research. Reflect and answer the following four questions:

1. *How do you understand reality?* Is there only one reality? Are there several realities? Are they all partial? Is reality a given? Or is it constructed? If so, how is it constructed?
2. *What do you believe about what can be known?* Must it be apprehended by the senses? Is it visible? Is it divisible? Is it external to people? Must it be fixed or static to apprehend?
3. *How do you think someone who wants to know should proceed?* Should he/she be involved? Should be separate from what are studying?
4. *What should the research method be like?* Identify some of the characteristics you think it should have to be truly scientific. For example: Objective? Replicable? Flexible?

Write down your answers and, as you progress through these articles, make the appropriate adjustments.
